# Not Seeing the Mites for the Hairs. Comment on Möth et al. Unexpected Effects of Local Management and Landscape Composition on Predatory Mites and Their Food Resources in Vineyards. *Insects* 2021, *12*, 180

**DOI:** 10.3390/insects12080671

**Published:** 2021-07-26

**Authors:** Peter Schausberger

**Affiliations:** Department of Behavioral and Cognitive Biology, University of Vienna, Althanstrasse 14, 1090 Vienna, Austria; peter.schausberger@univie.ac.at

## 1. Synopsis

Möth et al. (2021) [1] investigated the effects of pest management regime (integrated vs. organic), and field and landscape elements on plant-inhabiting mite abundance and pollen deposition in vineyards. One of their main conclusions, dubbed “unexpected”, is higher predatory mite abundance and pollen deposition in integrated rather than organic vineyards. Möth et al. (2021) [1] sampled leaves from highly diverse grape varieties, however, varieties and associated variability in mite microhabitat quality, which is largely determined by leaf surface morphology, were neglected in sampling plans and analyses. However, leaf surface morphology is a decisive factor for the occurrence and abundance of grape-inhabiting mites. Retrospectively scrutinizing the diversity and heterogeneity of sampled varieties and associated differences in leaf morphological characteristics, that is, hairiness of the leaf underside, revealed higher microhabitat quality in vineyards subjected to integrated pest management than in those subjected to organic pest management. Integrating leaf hairiness as an explanatory variable into analyses challenges the claims by Möth et al. [1] and puts the “unexpected” into perspective. Erect hair grades of the leaf underside had strong explanatory power and significantly influenced three of four dependent variables, that is, abundance of phytoseiid and eriophyid mites and pollen deposition on leaves. Re-analysis suggests only weak effects of pest management regimes. These effects are further undermined when considering that increased hairiness of the leaf underside may exert inter-related additive or synergistic effects on mite abundance, due to hampering pesticide deposits, increased provisioning of shelters, enhanced trapping of air-borne mites, and mite and pollen retention on leaves. I conclude that non-consideration of microhabitat quality may blur causal relationships between the occurrence of plant-inhabiting target organisms and larger-scale environmental variables.

## 2. Background

Möth et al. (2021) [1] investigated the influence of integrated versus organic pest management regimes as well as field and landscape elements on mite abundance and pollen deposition in Eastern Austrian vineyards. They examined the relevance of environmental factors at various spatial scales such as interrow vegetation type and cover at the field scale, and landscape scale factors such as surrounding vineyards and forests on mite abundance and pollen deposition on grape leaves. Pest management regimes were characterized by various sub-variables such as frequency and toxicity of the applied products. The main conclusions by Möth et al. (2021) [1], dubbed “unexpected”, is higher abundance of phytoseiid mites, primarily *Typhlodromus pyri*, and tydeid mites, primarily *Tydeus goetzi*, and higher pollen deposition in integrated than organically pest-controlled vineyards. Möth et al. (2021) [1] explain their findings by a mite-friendlier and more pollen favorable integrated pest management regime because of fewer sprays and lower toxicity of the applied products. Mite-promoting field and landscape factors put forward were a high percentage of vineyards surrounding the target (sampled) vineyard and spontaneous as compared to seeded interrow vegetation. Lacking report of the sampled grape varieties and associated lacking consideration of the microhabitat quality of the leaves of the sampled vineyards prompted writing this comment. Grape varieties strongly differ in leaf surface morphology and thus microhabitat quality for plant-inhabiting mites. Leaf surface morphology is generally acknowledged to be a decisive factor for the occurrence and abundance of plant-inhabiting arthropod, especially mite, populations and pollen deposition [2,3,4].

The target animals sampled by Möth et al. (2021) [1], plant-inhabiting mites, have body lengths of 0.2 (for eriophyid mites) to 0.5 mm (for phytoseiid mites) [5]. During the vegetative periods, the microhabitats of these mites are the leaves, and here, mainly the undersides. These animals thus primarily experience the leaf surfaces as their landscapes [6], in which they forage, reproduce and interact with other organisms. As with most ecological processes and patterns, scale is a critical issue when investigating environmental factors influencing the occurrence and abundance of plant-inhabiting mites in local fields. For plant-inhabiting mites, relevant spatial scales range from the microscale (sites within leaves to whole leaves) over intermediate scales such as whole plants to other plants within the vineyard, to the macroscale such as vegetation surrounding the vineyard and other local and regional landscape elements. Of primary importance, no matter of the ecological scale, are the microhabitats where the target animals, plant-inhabiting mites, primarily live and spend most of their lives, because some leaves provide microhabitats that are suitable for colonization, establishment and growth of their populations whereas others do not. If the leaf microhabitat is unsuitable, the surrounding field and landscape elements may be highly favorable for mites dispersing into the sampled sites yet the organisms in questions will not settle and reproduce, and as populations grow there [7]. In general, when developing sampling protocols and experimental designs for field and landscape studies, and deciding which factors should be included in analysis, the experimenter should project one’s view of the world into the target organism [8,9]. It has been stressed repeatedly that understanding of large-scale ecological processes and patterns is critically enhanced by mechanistic understanding of individual and population level processes at smaller spatial scales, such as the microhabitat level [8,10]. For the animals and study in question [1], if the leaf features determining microhabitat quality from a mite-centered perspective are not accounted for, blurred or erroneous conclusions about the influence of pest management, and larger spatial scale factors at the field and landscape levels on those mites may be drawn.

The sampling unit by Möth et al. (2021) [1] was 20 leaves per vineyard but the sampled vineyards consisted of 10 highly diverse grape varieties that were not homogenously distributed between integrated and organic vineyards (Möth, personal communication, Table S1). Thirteen of the 16 pairs of integrated and organic vineyards within a predefined radius of investigation had heterogeneous varieties and three had homogeneous varieties (Table S1). There is ample evidence that grape leaves differ in their quality as mite microhabitat among grape varieties [3,4]. For predatory mites, numerous studies have shown that one highly critical factor is the leaf surface morphology, and here, especially the occurrence and density of trichomes, bristles and domatia-like structures on the abaxial side of the leaf [11,12]. The dominant predatory mite species in vineyards of central Europe including Austria is *T. pyri*, which is known to thrive on grape varieties with pubescent and/or domatia-rich leaf undersides [13,14,15]. If the encountered microhabitat features are unfavorable, *T. pyri* does not successfully colonize and establish, and their populations do not grow, on those plants [16]. In consequence, and at a larger spatial scale, species-specific adaptations to leaf microhabitat features may even result in two adjacent vineyards of different varieties being populated by different predatory mite species or sustaining largely differing predatory mite abundances [7,13,14,17,18].

Based on the diversity and heterogeneous distribution of grape varieties within and between pairs of integrated and organic vineyards sampled by Möth et al. (2021) [1], I hypothesized that (i) grape variety-associated leaf morphological traits, i.e., hairiness of the leaf underside, differed between vineyards under organic and integrated pest management regimes, (ii) hairiness of the leaf underside has major explanatory power for the observed patterns of mite abundance and pollen deposition in Eastern Austrian vineyards, and (iii) addition of leaf hairiness as an independent variable in statistical modeling changes the set of variables that best explain mite abundance and pollen deposition. To test these hypotheses, the typical hairiness grades of the leaves of the grape varieties [19] sampled by Möth et al. (2021) [1] were compared between integrated and organic vineyards. Subsequently, some of the findings by Möth et al. (2021) [1] on mite abundance and pollen deposition in vineyards, as affected by pest management regime, field and landscape factors, arewere re-analyzed by additionally taking variety-specific hairiness of the leaf underside into account.

## 3. Methods

### 3.1. Characterization and Quantification of Microhabitat Quality 

Information on the grape varieties sampled by Möth et al. (2021) [1] were provided by Stefan Möth (personal communication; publicly accessible at https://doi.org/10.5281/zenodo.4562219, accessed on 12 March 2021). Grape cultivar-specific ampelographic characteristics of the leaf underside, that is, hairiness of the leaf blade and main veins, were derived from the OIV grape variety catalogue [19]. Hairiness grades are genetically-determined, variety-specific leaf morphological traits and well reflect the suitability of grape leaves for predatory mite occurrence and abundance [12]. In ampelography, leaf trichomes are divided into prostrate and erect (bristle) hairs on the leaf blade and on the main veins; their density grades may range from 1 (none or very sparse) to 9 (very dense) [19]. For varieties with a range of possible hairiness grades (Chardonnay, Cabernet Sauvignon and Sauvignon Blanc) in the OIV catalogue [19], the characterization followed the specifications for the variety type dominating in Austrian vineyards [20]. Grades for young leaves were used for the first sampling date in May and grades for mature leaves were used for the four later sampling dates. Separate grades for prostrate and erect (bristle-formed) hairs on the blade and main veins were summed up to create one overall grade (on blade plus on main veins) for prostrate hairs and one overall grade for erect hairs, jointly characterizing the leaf underside of a given variety. Erect (bristle) hairs are also a suitable indicator for the occurrence of acarodomatia-like structures on the leaf undersides [12] albeit domatia are poorly developed in cultivated *Vitis* species compared to their wild ancestors [11,21,22]. For prostrate hairs, grades 12 and 14 consisted of just 3 and 4 samples and were therefore included in grade 10 for analyses. 

### 3.2. Statistical Analyses

Statistical analyses were carried out using SPSS 26 (IBM, Armonk, NY, USA). 

Erect and prostrate hair grades of the undersides of young and mature leaves were compared between vineyards subjected to integrated and organic pest management regimes by Mann–Whitney U-tests. 

Erect and prostrate hair grades of the undersides of leaves were added as explanatory variables to the generalized linear models (GLMs) reported by Möth et al. (2021) [1]. Different from [1], both Akaike’s Information Criterion corrected for small sample size (AICC) and the Bayesian Information Criterion (BIC) were used to identify the best-fitting, i.e., the most parsimonious, model having the fewest parameters. The reason for additional use of BIC is that BIC penalizes more complex models more strongly than does AICC, i.e., it is more stringent regarding parsimony [23]. Starting with the full models for each dependent variable, explanatory variables were subsequently removed from the models in a stepwise procedure until arriving at the most parsimonious model providing the best fit according to AICC and/or BIC (difference >2 either for AICC and/or BIC to the next best model). Similarly, as in [1], lg(x + 1) transformed data of phytoseiid, tydeid and eriophyid mites per 100 cm^2^ leaf area and pollen density per 1 cm^2^ leaf area were used as dependent variables, percent vineyards in the surrounding, proportion of total and woody semi-natural habitats (SNHs), season-specific interrow cover percentage (spring-specific cover for the first date in May, and summer-specific cover for the other four dates) and pollen availability (lg(x + 1); only for mites), were used a covariates (continuous variables); pest management type (integrated versus organic), interrow vegetation type (poor, rich, spontaneous), and grape variety-specific prostrate and erect hair grades were used as nominal (categorical) variables. The hair grades were considered ordinal but not continuous variables because the intervals between grades are only an approximation of increasing intensity of hairiness of the leaf undersides but do not exactly and non-linearly reflect the proportional blade and main vein areas covered by hairs. Different from [1], phytoseiid abundance was not included as covariate in the GLM on their prey, eriophyid mites. The reason is that predator-prey relations are typically non-linear. Due to the time delay in numerical response of predators to the abundance dynamics of their prey, predator-prey population curves often oscillate in a sinus-like fashion with a time lag between prey and predators. Thus, depending on the sampling point in these cycles, all sorts of relation of predator-prey abundances, from negative to neutral to positive, may be observed.

## 4. Results

**Figure 1:**
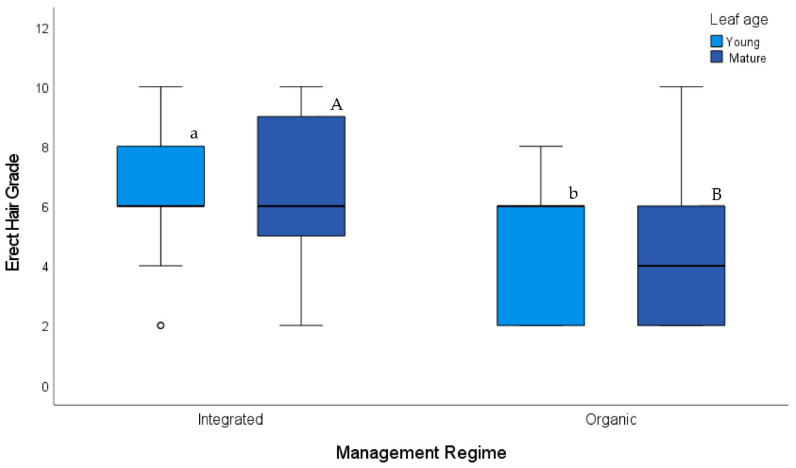
Variety-dependent erect hair grades (representing the sum of grades on the blade and main veins) of the leaf underside in vineyards under integrated and organic pest management regimes sampled by [1]. Thick horizontal lines represent the median, boxes represent the interquartile (IQ) range, whiskers are 1.5 times the IQ range, symbols depict the outliers. Different small and capital letters on top of boxes indicate significant differences in leaf hairiness of sampled vineyards under integrated and organic pest management (*n* = 16 for each pest management regime and leaf age category; Mann–Whitney U-tests *p* < 0.05).

The ten grape varieties sampled by Möth et al. (2021) [1] largely varied in erect and prostrate hair grades on the leaf undersides and variety pairs under integrated and organic pest management only matched in 3 of 16 circles (Table S1). Erect hair grades of the leaf underside differed significantly between vineyards subjected to integrated and organic pest management (Mann–Whitney U-tests; *n* = 16 each for integrated and organic vineyards; U = 71.5, *p* = 0.022 for erect hair grades of young leaves and U = 65.5, *p* = 0.015 for mature leaves); vineyards under integrated pest management had more erect hairs on the underside of both young and mature leaves than vineyards under organic pest management (Figure 1). Prostrate hair grades of young (U = 117, *p* = 0.665) and mature (U = 115, *p* = 0.642) leaves were similar in both types of vineyards. 

**Figure 2:**
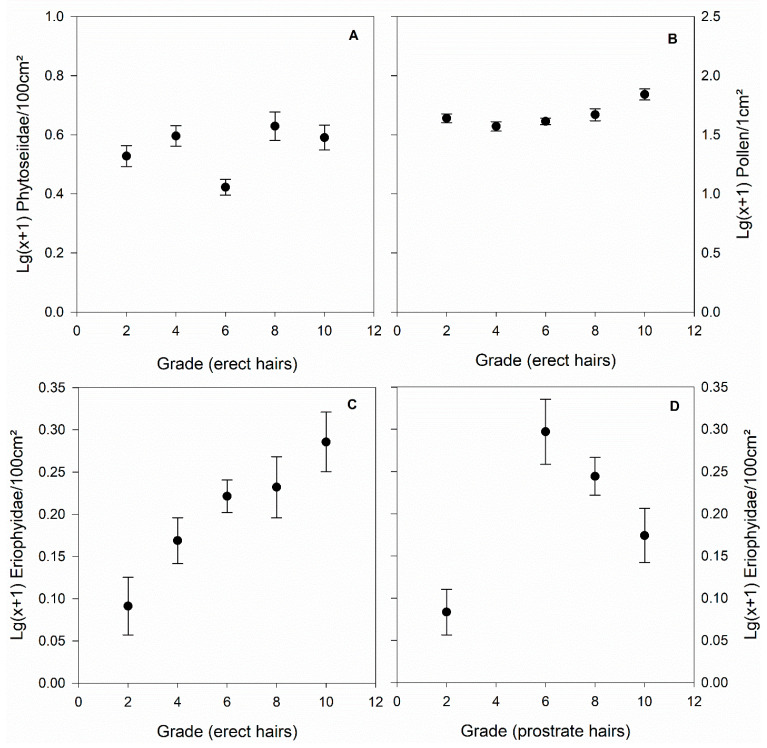
Significant influence of erect and prostrate hair grades of the leaf underside on phytoseiid (**A**) and eriophyid (**C**,**D**) abundance and pollen deposition (**B**) (mean ± SE) in vineyards sampled by [1]. Figures visualizing all significant explanatory variables in GLMs on phytoseiid, tydeid and eriophyid abundance as well as pollen deposition are provided in the Supplementary File (Figures S1a,b, S2a,b, S3 and S4).

The most parsimonious GLMs (Table 1) revealed that date (for all four dependent variables; Figures S1a, S2a, S3 and S4), erect hair grades of the leaf underside (for phytoseiid and eriophyid mite abundance and pollen deposition; Figure 2), and interrow vegetation type (for phytoseiid, eriophyid and tydeid mite abundance; Figures S1a, S2a and S3) were the most relevant predictors across all four dependent variables. Type of pest management (Figure S1a) and percent vineyards in the surrounding of the target vineyard (Figure S1b) had only an influence on phytoseiid mite abundance, prostrate hair grade of the leaf underside influenced only eriophyid mite abundance (Figure 2) and pollen deposition on leaves was a significant covariate only for eriophyid mite abundance (Figure S2b). The second best-fitting model for pollen deposition (difference <2 to the best-fitting model, that is, 1.201 in BIC and 1.261 in AICC) also included distance to the next woody semi-natural habitat, however, for the sake of parsimony, only the simplest model with the lowest AICC and BIC values is presented here. For the other three dependent variables, the second-best models had >2 difference in AICC and/or BIC. 

## 5. Discussion

Accounting for leaf surface traits largely determining grape leaf microhabitat quality for plant-inhabiting mites, i.e., grading of the density of two types of hairs (erect and prostrate) on the leaf underside, critically changed the set of significant pest management, field and landscape factors affecting phytoseiid mites, eriophyid mites and pollen deposition in vineyards sampled by [1]. Higher erect hair grades were associated with higher phytoseiid and eriophyid mite densities and pollen deposition on leaves. The only landscape- scale factors remaining significant, relative to the analysis by [1], are percent vineyards in the surrounding of the target vineyard positively affecting phytoseiid mite abundance and, with lower importance because only present in the second best-fitting model, distant to the next woody semi-natural habitat for pollen deposition. Pest management regime only influenced phytoseiid mites, with lower densities in organic than integrated vineyards, whereas non-consideration of microhabitat quality in analyses prompted Möth et al. (2021) [1] to conclude on significant pest management influences on phytoseiid and tydeid mite abundance and pollen deposition on leaves.

Differences in leaf morphological features, such as degree of hairiness and/or occurrence of domatia-like structures on the underside of leaves, have important implications to the effects of pest management, and field and landscape factors on mite occurrence and abundance and pollen deposition. The re-analyses presented here did not include interactions between leaf hairiness and other explanatory variables, to make it better comparable to the analyses reported by [1], but data exploration suggested some significant interactions. For example, more favorable hairiness of the leaf underside and more favorable spontaneous interrow vegetation were both biased towards vineyards under integrated pest management and the positive effect of leaf hairiness on phytoseiid mite abundance was more evident in vineyards with spontaneous versus seeded interrow vegetation. Moreover, one has to be aware that jointly biased explanatory variables may have additive or even synergistic effects, aggravating identification of cause and effect. Most importantly, leaf morphological structures such as hairiness and domatia also heavily influence the effects of pesticide sprays because such structures provide shelters and refuges protecting the mites from direct exposure to sprays and/or contact with their residues [3]. Additionally, hairy leaf surfaces are more hydrophobic than smooth leaves (hairy leaves have higher contact angles for fluid droplets [23]) resulting in poorer spread, adhesion to, and penetration of, the leaf tissue, and decreased residues of sprays, leading to more likely and longer lasting contact with sprays by mites living on smooth leaves [24,25]. This may partially explain why Möth et al. (2021) [1] did not detect a direct significant influence of the toxicity of the applied products in their best-fitting GLMs explaining mite abundance. Nonetheless, the potentially detrimental effects of frequent sulfur spray applications, as sometimes conducted in organic viticulture, on predatory mites have long been known [26,27] yet such adverse effects may especially apply to varieties with smooth surfaces. Leaf hairiness also influences pollen deposition on leaves because hairs serve as traps for air-borne pollen grains and enhance retention of pollen grains on the leaf surface [3]. Hairiness effects such as providing protection and enhanced trapping of air-borne mites, also apply to eriophyid mites. Regarding eriophyid mites, it is noteworthy mentioning that the washing technique employed by [1] is little suitable for sampling gall-inducing and gall-inhabiting eriophyid mites *Colomerus vitis* because of the contents of the galls, where the eriophyids primarily live, is barely accessible to the washing technique. Retrospectively, counting the number of galls per leaf, opening some galls and counting the number of gall-inhabiting eriophyids would have yielded a more realistic picture of eriophyid occurrence and abundance.

## 6. Conclusions

In an ideal way, albeit possibly difficult to achieve in such a field study, Möth et al. (2021) [1] would have sampled paired vineyards of the same variety within circles. Alternatively, and more easily achievable, they could have sampled pairs of different varieties that have similar leaf surface structures. At the very least, they should have counterbalanced widely-known issues associated with variety heterogeneity by including different varieties with similar leaf hairiness grades in each pest management regime, integrated and organic, to buffer any presumable influences of variety-specific microhabitat qualities for mite population development and pollen deposition. Biased distribution of varieties and not accounting for mite microhabitat quality may have blurred cause and effect. Given the numerous positive effects of leaf hairiness on pollen deposition, microhabitat quality for plant-inhabiting mites, and poorer adhesion of pesticides, combined with more favorable interrow vegetation type (spontaneous), one may even speculate that the differences in phytoseiid mite abundance have causally little to do with the pest management regimes per se but are a consequence of the combination of various factors being favorably biased towards vineyards under integrated pest management. Overall, this comment emphasizes the need to assume a target-organism-centered view when developing field sampling plans for plant-inhabiting arthropods and provides a cautionary tale of the relevance of microhabitat quality in pertinent field and landscape-scale studies.

## Figures and Tables

**Table 1:** Results of generalized linear models (GLM; normal distribution, identity link) on phytoseiid, tydeid and eriophyid mite abundance per 100 cm^2^ leaf area and pollen deposition per 1 cm^2^ leaf area in vineyards sampled by Möth et al. (2021). All dependent variables were lg(x + 1) transformed before analysis. AICC = −58.118, BIC = −17.962 for Phytoseiidae; AICC = −215.227, BIC = −169.828 for Eriophyidae; AICC = −90.554, BIC = −66.906 for Tydeidae; AICC = −22.144, BIC = 7.132 for pollen. Significant explanatory variables of leaf hairiness are highlighted in bold.

Dependent and Explanatory Variables	Wald ꭓ^2^	df	*p* Value
**Phytoseiidae**			
(Intercept)	52.344	1	<0.001
Date	77.757	4	<0.001
Pest Management Regime	19.055	1	<0.001
Interrow Vegetation Type	8.618	2	0.013
**Erect Hair Grade**	**26.979**	**4**	**<0.001**
Percent Vineyards	22.815	1	<0.001
**Eriophyidae**			
(Intercept)	0.858	1	0.354
Date	81.995	4	<0.001
Interrow Vegetation Type	7.229	2	0.027
**Prostrate Hair Grade**	**21.346**	**3**	**<0.001**
**Erect Hair Grade**	**15.254**	**4**	**0.004**
Pollen	3.858	1	0.050
**Tydeidae**			
(Intercept)	184.144	1	<0.001
Date	138.225	4	<0.001
Interrow Vegetation Type	25.281	2	<0.001
**Pollen**			
(Intercept)	8420.283	1	<0.001
Date	319.278	4	<0.001
**Erect Hair Grade**	**23.057**	**4**	**<0.001**

## Data Availability

The data presented in this study are available in the supplementary materials; the data presented by [1] are openly available in Zenodo at: http://doi.org/10.5281/zenodo.4450163, accessed on 12 March 2021.

## Supplementary Materials

The following are available online at https://www.mdpi.com/article/10.3390/insects12080671/s1. Table S1: Erect and prostrate hair grades of the 32 vineyards paired within 16 circles, each having a 500 m radius, sampled by Möth et al. (2021) [1]. Erect and prostrate hair grades of the leaf blade and the main veins given in the OIV catalogue [19] were summed (minimum sum is 2, maximum is 18); in case of a variety-typical range of grades given in [19], the specifications of [20] were additionally considered to select the best matching grades for this variety in Austria. Due to rarity of cases in prostrate hair grades 12 and 14, these cases were included in grade 10 for analyses, Figure S1: Explanatory variables (1.a nominal variables (mean ± SE): (A) date, (B) erect hair grade of the leaf underside, (C) vegetation type, (D) pest management regime; 1.b continuous variable (linear regression ± 95% CI): percent vineyards in the surrounding of the target vineyard) significantly influencing phytoseiid mite abundance in vineyards, Figure S2: Explanatory variables (2.a nominal variables (mean ± SE): (A) date, (B) vegetation type, (C) erect hair grade of the leaf underside, (D) prostrate hair grade of the leaf underside; 2.b continuous variable (linear regression ± 95% CI): percent vineyards in the surrounding of the target vineyard) significantly influencing eriophyid mite abundance in vineyards, Figure S3: Explanatory variables: (A) date, (B) vegetation type significantly influencing tydeid mite abundance in vineyards, Figure S4: Explanatory variables: (A) date, (B) erect hair grade on the leaf underside significantly influencing pollen deposition in vineyards.

## References

[1] Möth S., Walzer A., Redl M., Petrović B., Hoffmann C., Winter S. (2021). Unexpected effects of local management and landscape composition on predatory mites and their food resources in vineyards. Insects.

[2] Price P.W., Bouton C.E., Gross P., McPheron B.A., Thompson J.N., Weis A.E. (1980). Interactions Among Three Trophic Levels: Influence of Plants on Interactions Between Insect Herbivores and Natural Enemies. Annu. Rev. Ecol. Syst..

[3] Schmidt R.A. (2013). Leaf structures affect predatory mites (Acari: Phytoseiidae) and biological control: A review. Exp. Appl. Acarol..

[4] Tixier M.-S. (2018). Predatory Mites (Acari: Phytoseiidae) in Agro-Ecosystems and Conservation Biological Control: A Review and Explorative Approach for Forecasting Plant-Predatory Mite Interactions and Mite Dispersal. Front. Ecol. Evol..

[5] Krantz G.W., Walter D.E. (2009). A Manual of Acarology.

[6] Walter D.E. (1996). Living on leaves: Mites, tomenta, and leaf domatia. Annu. Rev. Entomol..

[7] Tixier M.-S., Kreiter S., Auger P. (2000). Colonization of vineyards by phytoseiid mites: Their dispersal patterns in the plot and their fate. Exp. Appl. Acarol..

[8] Wiens J.A., Stenseth N.C., Van Horne B., Ims R.A. (1993). Ecological Mechanisms and Landscape Ecology. Oikos.

[9] Jackson H.B., Fahrig L. (2012). What size is a biologically relevant landscape?. Landsc. Ecol..

[10] Landsman A.P., Ladin Z.S., Gardner D., Bowman J.L., Shriver G., D’Amico V., Delaney D.A. (2019). Local landscapes and micro-habitat characteristics are important determinants of urban–suburban forest bee communities. Ecosphere.

[11] English-Loeb G., Norton A.P., Walker M.A. (2002). Behavioral and population consequences of acarodomatia in grapes on phytoseiid mites (Mesostigmata) and implications for plant breeding. Entomol. Exp. Appl..

[12] Loughner R., Goldman K., Loeb G., Nyrop J. (2008). Influence of leaf trichomes on predatory mite (*Typhlodromus pyri*) abundance in grape varieties. Exp. Appl. Acarol..

[13] Duso C. (1992). Role of *Amblyseius aberrans* (Oud.), *Typhlodromus pyri* Scheuten and *Amblyseius andersoni* (Chant) (Acari, Phytoseiidae) in vineyards. III. Influence of variety characteristics on the success of *A. aberrans* and *T. pyri* releases. J. Appl. Entomol..

[14] Duso C., Vettorazzo E. (1999). Mite Population dynamics on different grape varieties with or without phytoseiids released (Acari: Phytoseiidae). Exp. Appl. Acarol..

[15] Roda A., Nyrop J., English-Loeb G. (2003). Leaf pubescence mediates the abundance of non-prey food and the density of the predatory mite *Typhlodromus pyri*. Exp. Appl. Acarol..

[16] Loughner R., Wentworth K., Loeb G., Nyrop J. (2010). Leaf trichomes influence predatory mite densities through dispersal behavior. Entomol. Exp. Appl..

[17] Camporese P., Duso C. (1996). Different colonization patterns of phytophagous and predatory mites (Acari: Tetranychidae, Phytoseiidae) on three grape varieties: A case study. Exp. Appl. Acarol..

[18] Tixier M.-S., Kreiter S., Auger P., Weber M. (1998). Colonization of Languedoc vineyards by phytoseiid mites (Acari: Phytoseiidae): Influence of wind and crop environment. Exp. Appl. Acarol..

[19] OIV-Organisation Internationale de la Vigne et du Vin (2009). Description of World Wine Varieties. https://www.oiv.int/en/technical-standards-and-documents/description-of-grape-varieties/description-of-grape-varieties-throughout-the-world.

[20] HBLA und Bundesamt für Wein- und Obstbau Klosterneuburg (2021). Rebsortenkatalog-Qualitätsweinsorten. http://www.weinobstklosterneuburg.at/service/rebsortenkatalog/qualitaetsweinrebsorten.html.

[21] Gerrath J., Posluszny U., Melville L. (2015). Taming the Wild Grape: Botany and Horticulture in the Vitaceae.

[22] Karban R., English-Loeb G., Walker M.A., Thaler J. (1995). Abundance of phytoseiid mites on *Vitis* species: Effects of leaf hairs, domatia, prey abundance and plant phylogeny. Exp. Appl. Acarol..

[23] Brewer M.J., Butler A., Cooksley S.L. (2016). The relative performance of AIC, AICC and BIC in the presence of unobserved heterogeneity. Methods Ecol. Evol..

[24] Konlechner C., Sauer U. (2016). Ultrastructural leaf features of grapevine cultivars (*Vitis vinifera* L. ssp. *vinifera*). OENO One.

[25] Xu L., Zhu H., Ozkan H.E., Bagley W.E., Krause C.R. (2011). Droplet evaporation and spread on waxy and hairy leaves associated with type and concentration of adjuvants. Pest Manag. Sci..

[26] Gadino A.N., Walton V.M., Dreves A.J. (2011). Impact of vineyard pesticides on a beneficial arthropod, *Typhlodromus pyri* (Acari: Phytoseiidae), in laboratory bioassays. J. Econ. Èntomol..

[27] Hanna R., Zalom F.G., Wilson L.T., Leavitt G.M. (1997). Sulfur can suppress mite predators in vineyards. Calif. Agric..

