# Supplementary material for: Not Seeing the Mites for the Hairs. Comment on Möth et al. Unexpected Effects of Local Management and Landscape Composition on Predatory Mites and Their Food Resources in Vineyards. Insects 2021, 12, 180
Source: Insects. 2021 Jul 26;12(8):671. doi: 10.3390/insects12080671 (PMC8396593; doi:10.3390/insects12080671)
Supplement: Supplementary file 1 [file insects-12-00671-s001.zip › insects-1180870-supplementary.pdf]

Supplementary files to

Not Seeing the Mites for the Hairs. Comment on Möth et al. Unexpected Effects of Local Management and Landscape Composition on Predatory Mites and Their Food Resources in Vineyards. *Insects* 2021, *12*, 180

Peter Schausberger*

Department of Behavioral and Cognitive Biology, University of Vienna, Althanstrasse 14, 1090 Vienna, Austria

*Correspondence: peter.schausberger@univie.ac.at

Table S1. Erect and prostrate hair grades of the 32 vineyards paired within 16 circles, each having a 500 m radius, sampled by Möth et al. (2021) [1]. Erect and prostrate hair grades of the leaf blade and the main veins given in the OIV catalogue [19] were summed (minimum sum is 2, maximum is 18); in case of a variety-typical range of grades given in [19], the specifications of [20] were additionally considered to select the best matching grades for this variety in Austria. Due to rarity of cases in prostate hair grades 12 and 14, these cases were included in grade 10 for analyses.

Circle	Vineyard ID	Variety	Pest management	Hair grade (leaf age)			
				Erect (young)	Prostrate (young)	Erect (mature)	Prostrate (mature)
1	AODc_1	Blauburger	Organic	2	8	2	8
2	AODc_2	Grüner Veltliner	Organic	6	10	4	8
3	AOLc_3	Chardonnay	Organic	2	6	4	6
4	AODc_4	Blaufränkisch	Organic	6	2	6	2
5	AOLc_5	Weissburgunder	Organic	2	6	2	6
6	AODc_6	Grüner Veltliner	Organic	6	10	4	8
7	AODc_7	Zweigelt	Organic	8	2	10	2
8	AODc_8	Blaufränkisch	Organic	6	2	6	2
9	AOLc_9	Weissburgunder	Organic	2	6	2	6
10	AODc_10	Chardonnay	Organic	2	6	4	6
11	AOLc_11	Welschriesling	Organic	6	8	6	8
12	AOLc_12	Welschriesling	Organic	6	8	6	8
13	AOLc_13	Weissburgunder	Organic	2	6	2	6
14	AOLc_14	Blaufränkisch	Organic	6	2	6	2
15	AODc_15	Blaufränkisch	Organic	6	2	6	2
16	AOLc_16	Blauburger	Organic	2	8	2	8
1	ACDc_1	Cabernet Sauvignon	Integrated	8	10	8	10
2	ACDc_2	Blaufränkisch	Integrated	6	2	6	2
3	ACDc_3	Blaufränkisch	Integrated	6	2	6	2
4	ACDc_4	Blaufränkisch	Integrated	6	2	6	2

5	ACLc_5	Cabernet Sauvignon	Integrated	8	10	8	10
6	ACLc_6	Zweigelt	Integrated	8	2	10	2
7	ACLc_7	Grüner Veltliner	Integrated	6	10	4	8
8	ACLc_8	Welschriesling	Integrated	6	8	6	8
9	ACLc_9	Grüner Veltliner	Integrated	6	10	4	8
10	ACDc_10	Zweigelt	Integrated	8	2	10	2
11	ACLc_11	Chardonnay	Integrated	2	6	4	6
12	ACLc_12	Sauvignon Blanc	Integrated	4	10	8	10
13	ACDc_13	Zweigelt	Integrated	8	2	10	2
14	ACDc_14	Blaufränkisch	Integrated	6	2	6	2
15	ACLc_15	Furmint	Integrated	10	10	10	10
16	ACDc_16	Blauburger	Integrated	2	8	2	8

Supplementary figures

Figure S1. Explanatory variables (**S1.a** nominal variables (mean ± SE) - (A) date, (B) erect hair grade of the leaf underside, (C) vegetation type, (D) pest management regime; **S1.b** continuous variable (linear regression ± 95% CI) - percent vineyards in the surrounding of the target vineyard) significantly influencing phytoseiid mite abundance in vineyards.

Figure S2. Explanatory variables (**S2.a** nominal variables (mean ± SE) – (A) date, (B) vegetation type, (C) erect hair grade of the leaf underside, (D) prostrate hair grade of the leaf underside; **S2.b** continuous variable (linear regression ± 95% CI) - percent vineyards in the surrounding of the target vineyard) significantly influencing eriophyid mite abundance in vineyards.

Figure S3. Explanatory variables – (A) date, (B) vegetation type - significantly influencing tydeid mite abundance (mean ± SE) in vineyards.

Figure S4. Explanatory variables – (A) date, (B) erect hair grade on the leaf underside - significantly influencing pollen deposition (mean ± SE) in vineyards.

Figure S1.a

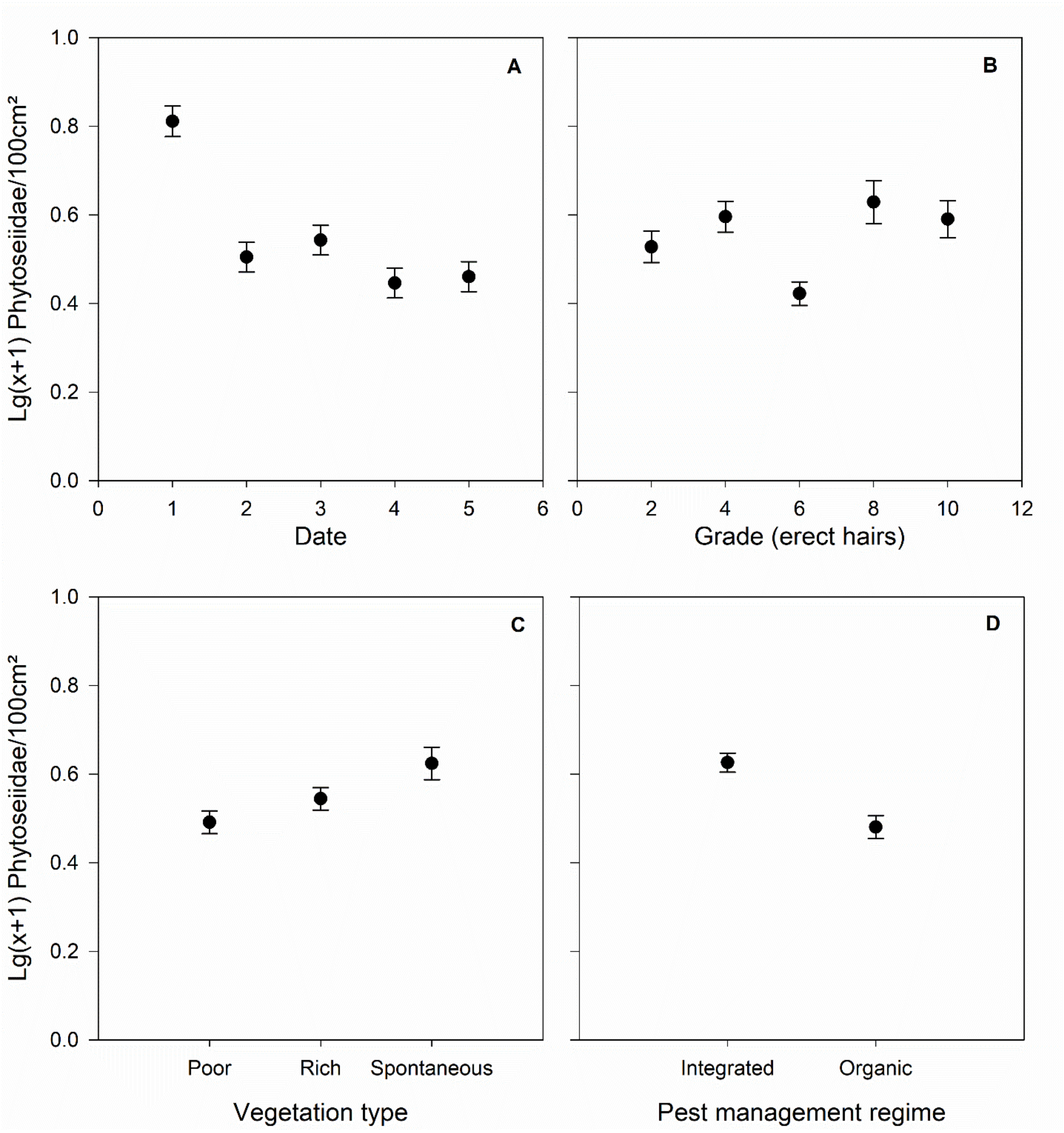

A
B
C
D
Lg(x+1) Phytoseiidae/100cm²
Lg(x+1) Phytoseiidae/100cm²
1.0
0.8
0.6
0.4
0.2
0.0
0
1
2
3
4
5
6
Date
0
2
4
6
8
10
12
Grade (erect hairs)
Poor
Rich
Spontaneous
Vegetation type
Integrated
Organic
Pest management regime

Figure S1.b

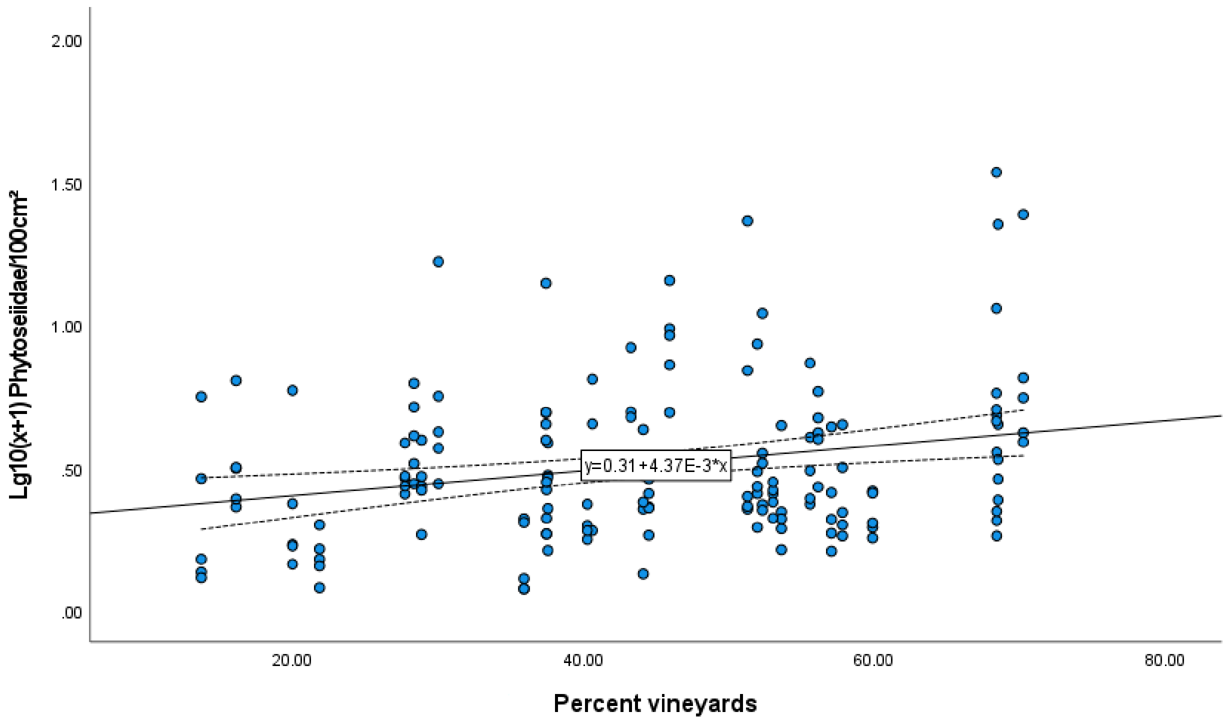

2.00
1.50
1.00
.50
.00
Lg10(x+1) Phytoseiidae/100cm²
y=0.31+4.37E-3*x
20.00
40.00
60.00
80.00
Percent vineyards

Figure S2.a

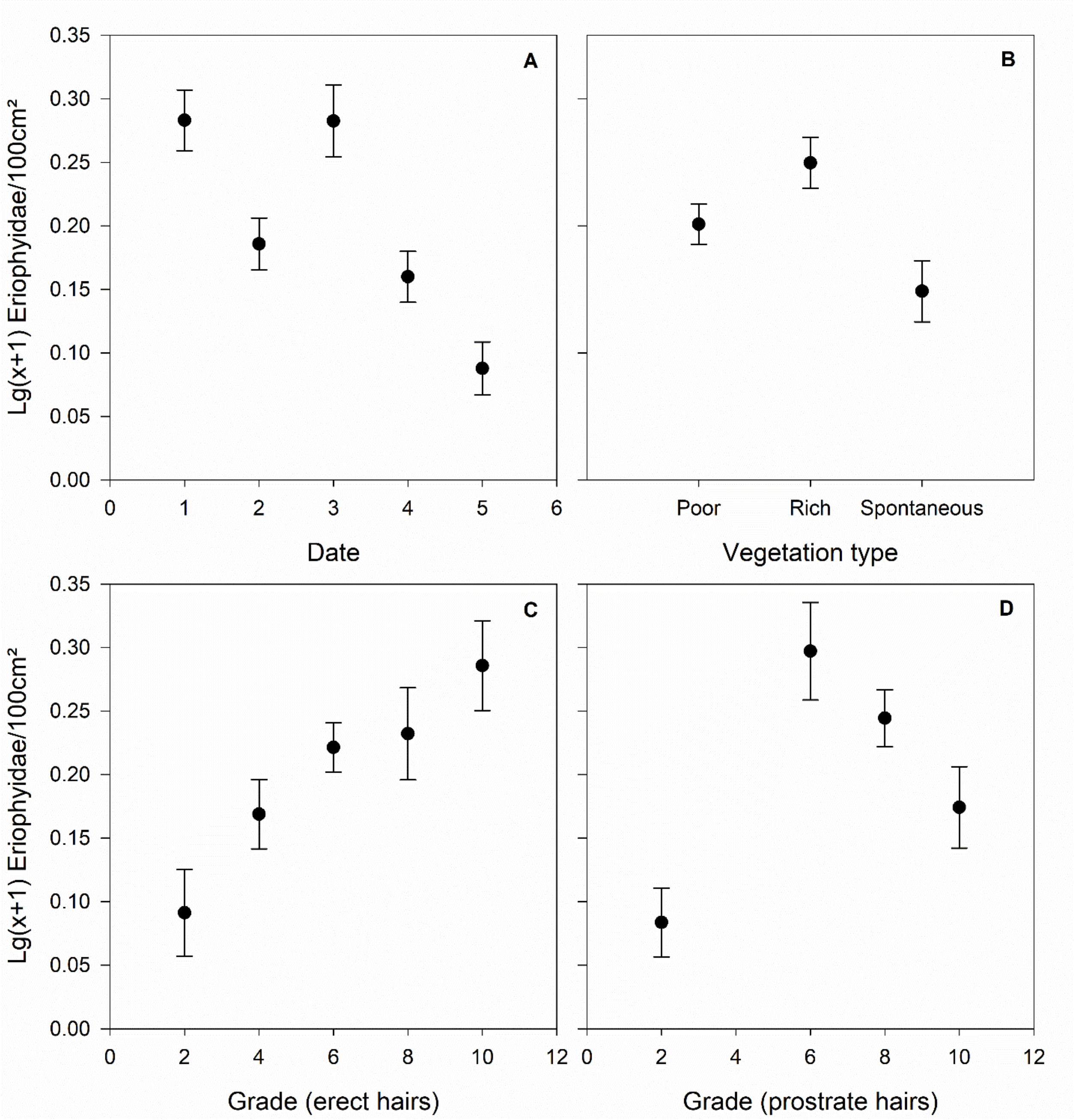

A
B
C
D
Lg(x+1) Eriophyidae/100cm²
0.35
0.30
0.25
0.20
0.15
0.10
0.05
0.00
0
1
2
3
4
5
6
Date
Poor
Rich
Spontaneous
Vegetation type
0
2
4
6
8
10
12
Grade (erect hairs)
Grade (prostrate hairs)

Figure S2.b

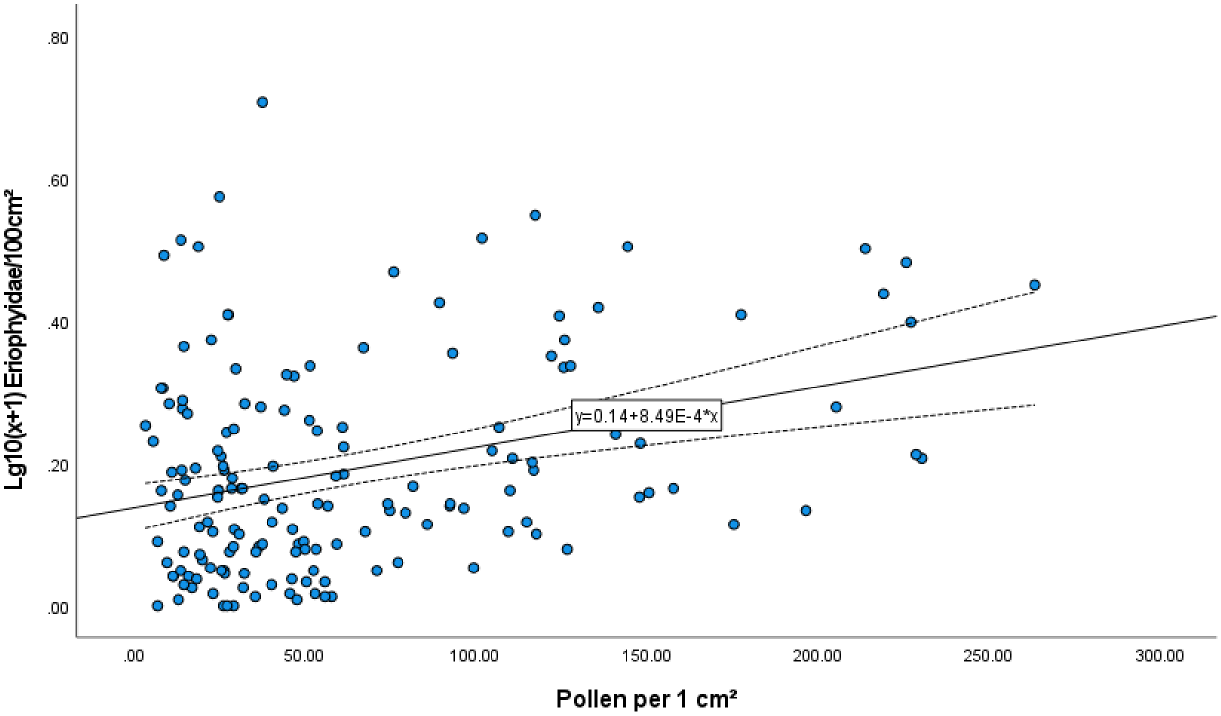
.80
.60
.40
.20
.00
Lg10(x+1) Eriophyidae/100cm²
y=0.14+8.49E-4*x
.00
50.00
100.00
150.00
200.00
250.00
300.00
Pollen per 1 cm²

Figure S3

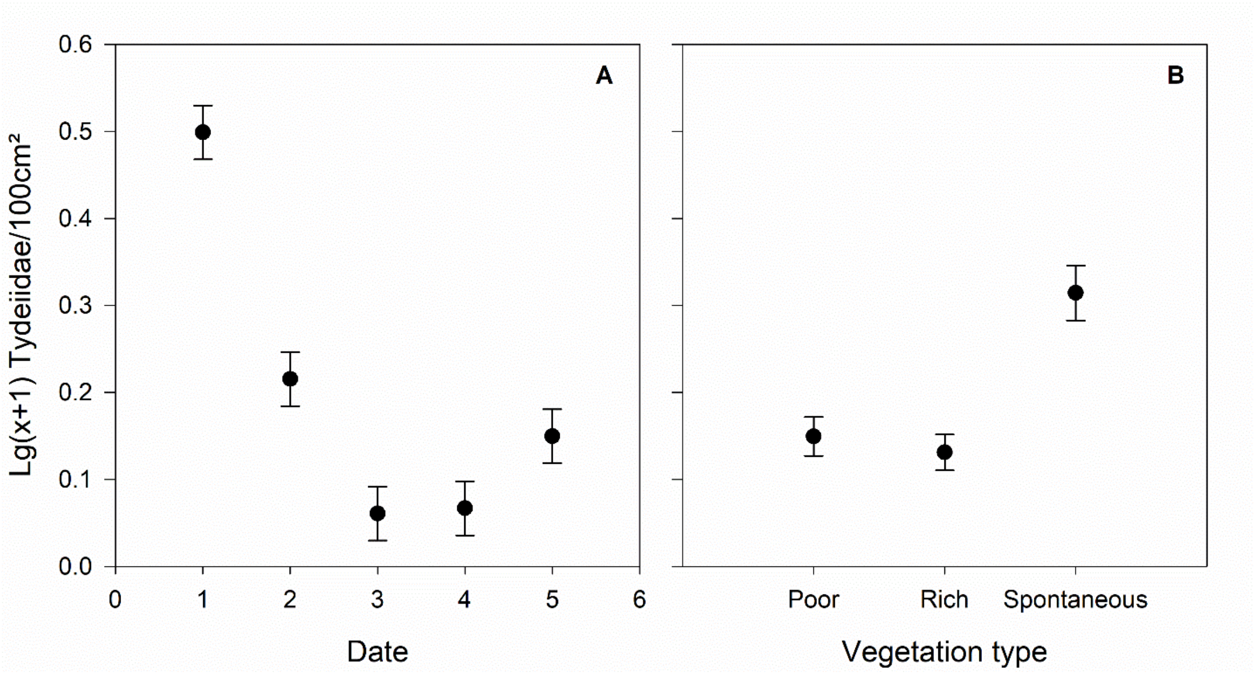

A
B
Lg(x+1) Tydeiidae/100cm²
0.0
0.1
0.2
0.3
0.4
0.5
0.6
0
1
2
3
4
5
6
Date
Poor
Rich
Spontaneous
Vegetation type

Figure S4

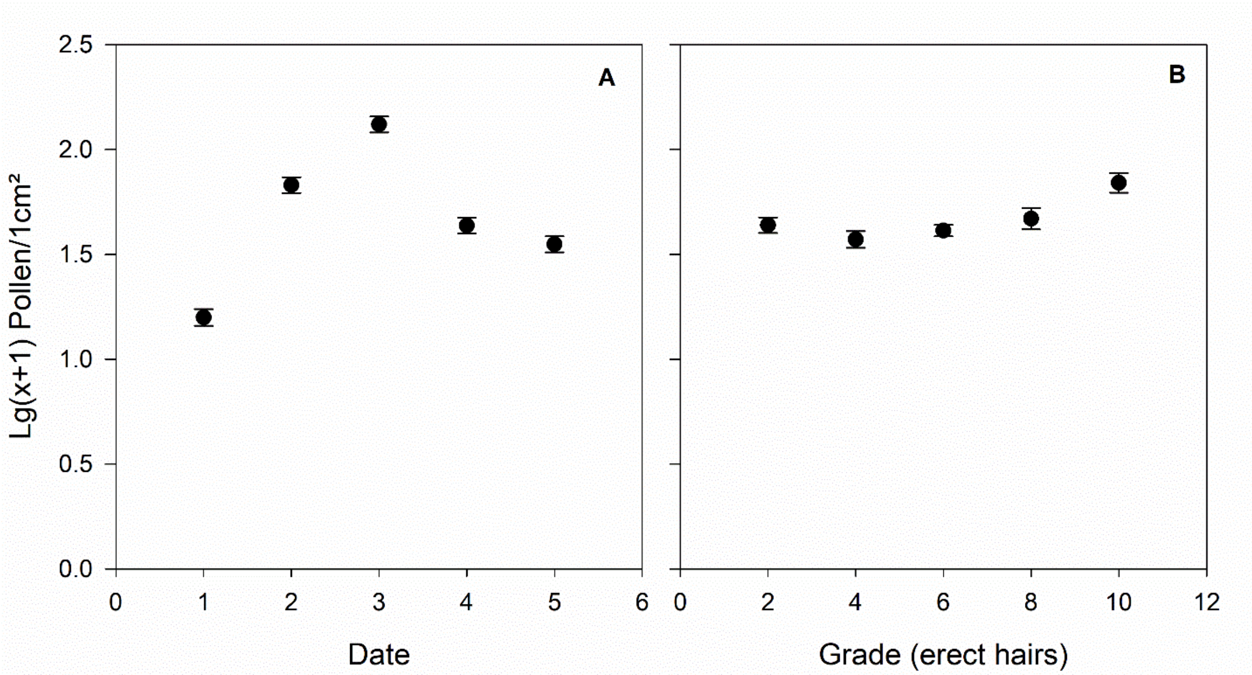
A
B
2.5
2.0
1.5
1.0
0.5
0.0
Lg(x+1) Pollen/1cm²
0
1
2
3
4
5
6
Date
0
2
4
6
8
10
12
Grade (erect hairs)
